# Correction: Power analysis for personal light exposure measurements and interventions

**DOI:** 10.1371/journal.pone.0325944

**Published:** 2025-06-02

**Authors:** Johannes Zauner, Ljiljana Udovicic, Manuel Spitschan

In the originally published article [1], two issues are identified in the references related to the CODECHECK initiative.

Reference 36 was incorrectly formatted [2]. The correct reference 36 is: Nüst D, Eglen SJ. CODECHECK: an Open Science initiative for the independent execution of computations underlying research articles during peer review to improve reproducibility. *F1000Research*. 2021;10(253). https://doi.org/10.12688/f1000research.51738.2 PMID: 34367614

A relevant reference was omitted. The citation to Eglen (2024) [3] should be included alongside reference 36 to acknowledge the specific CODECHECK certificate associated with the article in the last sentence of the ‘Software’ subsection of ‘Materials and methods’. The correct sentence is:

*All scripts and results are part of S1 File as an HTML Quarto document [*35*], including the necessary source code for replication. Reproducibility is ensured through CODECHECK [*36*](Eglen, 2024).*

These references [2,3] provide both general context for the CODECHECK process and specific documentation of the independent verification conducted for this article. The authors affirm the importance of reproducibility, transparency, and appropriate citation of tools and services that support rigorous scientific research.
