# Supplementary material for: Power analysis for personal light exposure measurements and interventions
Source: PLoS One. 2024 Dec 11;19(12):e0308768. doi: 10.1371/journal.pone.0308768 (PMC11633969; doi:10.1371/journal.pone.0308768)
Supplement: S1 File — (HTML) [file pone.0308768.s001.html]

Power analysis for personal light exposure measurements and interventions


## Table of contents

- Preface
  - Analysis Outline
- Import and Data Preparation
  - Seed
  - Import
  - Preparing the Data
  - Metrics
  - Bootstrapping
  - Results

# Power analysis for personal light exposure measurements and interventions

 Code

- Show All Code
- Hide All Code
- ---
- View Source

Supplementary Document S1 - Statistical Analysis

Author

Johannes Zauner, Ljiljana Udovicic, Manuel Spitschan

## Preface

This is a supplementary document to the main manuscript of the same title. The following statistical analysis evaluates the necessary sample size to significantly detect a difference in metrics derived from wearable light logger data. The data were provided by BAuA and were collected as part of a study1 on evaluating personal light exposure between London and Dortmund.

The goal of this analysis is to provide a sensible sample size for a field test study of similar design. Data analysis will be performed in `R` statistical software, using the `LightLogR` R package, which is part of the MeLiDos project.

Code

```
#collect all the packages that are needed for the analysis
packages <- c("quarto", "tidyverse", "broom", "cowplot", "gt", "gtsummary", 
              "gghighlight", "patchwork", "readxl", "lme4", "lmerTest",
              "ggridges", "osfr")
packages_github <- c("tscnlab/LightLogR", "steffenhartmeyer/lightdosimetry")

#check if packages are installed, if not install them
if(!require(pacman)) {
  install.packages("pacman") 
  library(pacman)
  }

p_load(packages, character.only = TRUE)
p_load_gh(char = packages_github)

#at the time of writing, lme4 might need to be reinstalled from source, due to
#an update of the Matrix package (https://stat.ethz.ch/pipermail/r-package-devel/2023q4/010054.html)
# oo <- options(repos = "https://cran.r-project.org/")
# install.packages("Matrix", type = "source")
# install.packages("lme4", type = "source")
# options(oo)
# library(lme4)
# library(lmerTest)

#major parameters for the analysis
Power_level <- params$Power_level
sign_level <- params$sign_level
valid_data_threshold <- params$valid_data_threshold
n_samples <-  params$n_samples
sample_size <- params$sample_size_min:params$sample_size_max
```

### Analysis Outline

The analysis will calculate the necessary sample size to reach a power level of 0.8 for a range of relevant metrics of personal light exposure. These metrics will be included in `LightLogR` by the time the manuscript is published, but are presently taken from the `lightdosimetry` package by Steffen Hartmeyer.

#### General info

To be included in the analysis, each participant-day needs to have a minimum of 80.00% valid, non-missing data. Non-missing data is evaluated by a regular sequence of measurement intervals (epochs) that are dominant to the data. Valid data is evaluated by a column of the data that indicates whether the device was worn or not.

The various metrics are calculated for each participant-day. This dataset will be the basis for the analysis.

#### Analysis

The analysis uses a `bootstrap` method, i.e., it resamples the data with replacement to generate a distribution of the metric of interest (1000 resamples). The distribution is generated for both the January and June data, and within participant. Each resampled dataset is tested with a `linear mixed-effect` model to test for a significant difference between the two seasons, within participants, for each given metric. The percentage of resamples that are significant are counted and used to calculate the power level.

The analysis is repeated for a range of sample sizes (min = 3, max = 50), and the sample size that reaches the required `Power Level` is recorded and reported.

## Import and Data Preparation

### Seed

Here we set a seed for the random number generator, to ensure reproducibility of the analysis.

Code

```
#set a seed for reproducibility
set.seed(params$seed)
```

### Import

This section will import all the data and provide an overview as well as first measures to check and secure the data quality. A total of 26 files are in the folder (13 participants, 2 seasons each). They are, however, not consistent in their encoding, so we need to import them separately and then combine them. Also, two of them are kept in excel-files, which also requires separate handling.

Code

```
#list all the Participant Id's
Participant.IDs <- c("WRC5", "WOL2", "WNC1", "SIM1", "MIH2", "MES8", "LIR1",
                     "HNN1", "DWM1", "D7EB", "CET8", "BLD3", "AET3")

N_participants <- length(Participant.IDs)

#list all the files
files <- list.files(path = "Data", pattern = "*.csv", full.names = TRUE)
files_xlsx <- list.files(path = "Data", pattern = "*.xlsx", full.names = TRUE)

#extract only files for the participants in the list, using stringr
files <- files[str_detect(files, str_c(Participant.IDs, collapse = "|"))]
files_xlsx <- files_xlsx[str_detect(files_xlsx, str_c(Participant.IDs, collapse = "|"))]

#general import settings for the data, as it is a german dataset
column_names <- c("Zeile", "Datum", "Zeit", "Status") #this is a sequence of column 
german_encoding <- c(2, 4, 6, 8, 11, 13, 14, 16, 22) #these are the files that use german encoding
int_encoding <- base::setdiff(1:24, german_encoding) #these are the files that use standard encoding
na_s <- c("", "NA", "kZ") #these are the NA strings in the files
auto.id <- "^\\d{5}_(.{4})" #this is the regex to extract the participant id
tz <- "Europe/Berlin" #this is the timezone of the data

#read in the files with german encoding
LLdata <- import$Actiwatch_Spectrum(files[german_encoding], 
                                    locale = locale(encoding="latin1"),
                                    tz = tz, na = na_s, auto.id = auto.id,
                                    column_names = column_names)
```

```
Successfully read in 218654 observations from Actiwatch_Spectrum-file
Timezone set is Europe/Berlin.
The system timezone is Europe/Berlin. Please correct if necessary!
Start: 2015-01-13 06:00:00
End: 2015-01-22 10:16:00
Timespan: 9.2 days
Observation intervals: 
  Id    interval.time     n pct  
1 AET3  30s           24208 100% 
2 CET8  30s           26432 100% 
3 D7EB  30s           23832 100% 
4 DWM1  30s           24260 100% 
5 HNN1  30s           23807 100% 
6 LIR1  30s           23815 100% 
7 MES8  30s           24195 100% 
8 WOL2  30s           24247 100% 
9 WRC5  30s           23849 100%
```

Code

```
#read in the files with english encoding
LLdata2 <- import$Actiwatch_Spectrum(files[int_encoding], 
                                    tz = tz, na = na_s, auto.id = auto.id,
                                    column_names = column_names
                                    )
```

```
Successfully read in 402988 observations from Actiwatch_Spectrum-file
Timezone set is Europe/Berlin.
The system timezone is Europe/Berlin. Please correct if necessary!
Start: 2015-01-22 06:00:00
End: 2015-06-30 14:26:00
Timespan: 159 days
Observation intervals: 
   Id    interval.time                n pct  
 1 AET3  30s                      21518 100% 
 2 BLD3  30s                      30401 100% 
 3 CET8  30s                      21541 100% 
 4 D7EB  30s                      30220 100% 
 5 DWM1  30s                      30468 100% 
 6 HNN1  30s                      24278 100% 
 7 LIR1  30s                      24252 100% 
 8 MES8  30s                      21603 100% 
 9 MIH2  30s                      59346 100% 
10 MIH2  10936080s (~18.08 weeks)     1 0%   
11 SIM1  30s                      33412 100% 
12 WNC1  30s                      62746 100% 
13 WNC1  11795850s (~19.5 weeks)      1 0%   
14 WOL2  30s                      21592 100% 
15 WRC5  30s                      21596 100%
```

Code

```
#read all excel files in and clean them up to be in comparable shape
LLdata3 <- 
  read_xlsx(
    files_xlsx[1], skip =  149, na = na_s, .name_repair = "universal",
    )[-1, ]
```

```
New names:
• `Status „Nicht am Handgelenk“` -> `Status..Nicht.am.Handgelenk.`
• `Weißes Licht` -> `Weißes.Licht`
• `Rotes Licht` -> `Rotes.Licht`
• `Grünes Licht` -> `Grünes.Licht`
• `Blaues Licht` -> `Blaues.Licht`
• `Schlaf/Wach` -> `Schlaf.Wach`
• `S/W-Status` -> `S.W.Status`
```

Code

```
LLdata4 <- 
  read_xlsx(
    files_xlsx[2], skip = 164, na = na_s, .name_repair = "universal"
    )[-1, ]
```

```
New names:
• `Status „Nicht am Handgelenk“` -> `Status..Nicht.am.Handgelenk.`
• `Weißes Licht` -> `Weißes.Licht`
• `Rotes Licht` -> `Rotes.Licht`
• `Grünes Licht` -> `Grünes.Licht`
• `Blaues Licht` -> `Blaues.Licht`
• `Schlaf/Wach` -> `Schlaf.Wach`
• `S/W-Status` -> `S.W.Status`
```

Code

```
LLdata_xlsx <- 
  list(LLdata3, LLdata4) %>% 
  map2(files_xlsx,
       \(file, filenames) {
         file %>% 
           mutate(file.name = 
                    filenames %>% basename() %>% tools::file_path_sans_ext(),
                  Id = str_extract(file.name, auto.id, group = 1),
                  Zeit = hms::as_hms(Zeit),
                  Datetime = as.POSIXct(paste(Datum, Zeit), 
                                        format = "%Y-%m-%d %H:%M:%S", tz = "UTC") %>% 
                    force_tz(tz),
                  Zeile = as.integer(Zeile),
                  across(
                    c(Status..Nicht.am.Handgelenk., Markierung, Schlaf.Wach, 
                      Intervallstatus), as.factor))
       }) %>% 
  list_rbind()

#combine the two datasets
LLdata <- join_datasets(LLdata, LLdata2, LLdata_xlsx)
rm(LLdata2, LLdata_xlsx, LLdata3, LLdata4)

#differentiate the datasets by Season
LLdata <- 
  LLdata %>%
  group_by(Id, Season = quarter(Datetime)) %>% 
  mutate(
    Season = case_when(Season == 1 ~ "Winter", Season == 2 ~ "Summer") %>% 
      factor(levels = c("Winter", "Summer")))

#get a general overview
Figure.1 <- 
  LLdata %>% gg_overview() + 
  labs(x = "Month", y = "Participant ID") +
  scale_x_datetime(date_breaks = "1 month", date_labels = "%b") +
  coord_cartesian(xlim = c(as.POSIXct("2015-01-09", tz = tz), NA)) +
  theme(axis.text=element_text(size=8),
        text = element_text(size = 10),
        axis.line = element_line(linewidth = 0.25),
        axis.ticks = element_line(linewidth = 0.25))
```

```
Scale for x is already present.
Adding another scale for x, which will replace the existing scale.
```

Code

```
Figure.1
```

Code

```
#save the figure
ggsave("Figures/Figure1.png", Figure.1, height = 8.5, width = 8.5, units='cm')
```

The import doesn’t suggest that there are any gaps in the dataset, but just to make sure, let us search for gaps in the data with the new grouping.

Code

```
#search for gaps (none are found)
LLdata %>% gap_finder()
```

```
No gaps found
```

Now lets go into a more comprehensive overview.

Code

```
#create and save a figure with an overview of the illuminance values
Figure.S1 <- 
LLdata %>% 
 gg_days(y.axis = Weißes.Licht, x.axis.breaks = waiver(), linewidth = 0.1,
   x.axis.format = waiver(), 
   y.axis.label = "Illuminance (lx)", x.axis.label = "Dates")+ 
  facet_wrap(Id ~ Season, scales = "free_x", ncol = 2, strip.position = "left") +
  theme(axis.text=element_text(size=6),
        text = element_text(size=8),
        strip.text = element_text(size=6),
        panel.spacing = unit(0.1, "lines"))

Figure.S1
```

Code

```
ggsave("Figures/FigureS1.png", Figure.S1, height = 20, width = 17, units='cm')
```

We can see some variation in the data, but nothing unexpected.

### Preparing the Data

There are several steps required before we can go into metrics calculation:

- We need to calculate the `melanopic EDI` (MEDI), as these are not part of the dataset. `BAuA` has provided a calibration function that is based on their measurements of the device.
- We need to filter for dates that are relevant for the analysis, i.e., that are `day-shifts`.

#### Calculating melanopic EDI

Code

```
#calculate melanopic EDI based on the BAuA calibration
LLdata <- LLdata %>% mutate(MEDI = (Grünes.Licht+ Blaues.Licht)*4.3/1.3262)
```

#### Filtering for Day-Shifts

Sadly, the days with day-shift is not part of the dataset, but there is a manually collected list with that information. The `filter_list` will collect these dates and we filter the data accordingly with the `filter_Datetime_multiple()` function.

Code

```
#gathering the dates for the filter
filter_list <- 
  list(
    #Winter
    #Consecutive Days
    list(start = "2015-01-14", end = "2015-01-16", 
         only_Id = quote(Id == "AET3" & Season == "Winter")),
    list(start = "2015-01-19", end = "2015-01-20", 
         only_Id = quote(Id == "HNN1" & Season == "Winter")),
    list(start = "2015-01-16", end = "2015-01-17", 
         only_Id = quote(Id == "LIR1" & Season == "Winter")),
    list(start = "2015-01-23", end = "2015-01-25", 
         only_Id = quote(Id == "MIH2" & Season == "Winter")),
    list(start = "2015-01-26", end = "2015-01-27", 
         only_Id = quote(Id == "WNC1" & Season == "Winter")),
    list(start = "2015-01-14", end = "2015-01-18",
         only_Id = quote(Id == "WRC5" & Season == "Winter")),
    list(start = "2015-01-28", end = "2015-01-29",
         only_Id = quote(Id == "SIM1" & Season == "Winter")),
    #Days with gaps
    list(start = "2015-01-14", end = "2015-01-20",
         filter.expr = quote(!(day(Datetime) %in% c(17, 18))),
         only_Id = quote(Id == "CET8" & Season == "Winter")),
    list(start = "2015-01-14", end = "2015-01-20",
         filter.expr = quote(!(day(Datetime) %in% c(17, 18))),
         only_Id = quote(Id == "DWM1" & Season == "Winter")),
    list(start = "2015-01-14", end = "2015-01-20",
         filter.expr = quote(!(day(Datetime) %in% c(17, 18))),
         only_Id = quote(Id == "D7EB" & Season == "Winter")),
    list(start = "2015-01-14", end = "2015-01-20",
         filter.expr = quote(!(day(Datetime) %in% c(17, 18))),
         only_Id = quote(Id == "MES8" & Season == "Winter")),
    list(start = "2015-01-23", end = "2015-01-29",
         filter.expr = quote(!(day(Datetime) %in% c(24, 25))),
         only_Id = quote(Id == "BLD3" & Season == "Winter")),
    list(start = "2015-01-14", end = "2015-01-20",
         filter.expr = quote(!(day(Datetime) %in% c(17, 18))),
         only_Id = quote(Id == "WOL2" & Season == "Winter")),
    #Summer
    #Consecutive Days    
    list(start = "2015-06-15", end = "2015-06-16", 
         only_Id = quote(Id == "AET3" & Season == "Summer")),
    list(start = "2015-06-11", end = "2015-06-12", 
         only_Id = quote(Id == "CET8" & Season == "Summer")),
    list(start = "2015-06-20", end = "2015-06-22", 
         only_Id = quote(Id == "DWM1" & Season == "Summer")),
    list(start = "2015-06-23", end = "2015-06-25", 
         only_Id = quote(Id == "D7EB" & Season == "Summer")),
    list(start = "2015-06-10", end = "2015-06-14", 
         only_Id = quote(Id == "HNN1" & Season == "Summer")),
    list(start = "2015-06-15", end = "2015-06-16", 
         only_Id = quote(Id == "LIR1" & Season == "Summer")),
    list(start = "2015-06-13", end = "2015-06-14", 
         only_Id = quote(Id == "MES8" & Season == "Summer")),
    list(start = "2015-06-10", end = "2015-06-12", 
         only_Id = quote(Id == "MIH2" & Season == "Summer")),
    #Days with gaps
        list(start = "2015-06-20", end = "2015-06-25",
         filter.expr = quote(!(day(Datetime) %in% c(21,22,23))),
         only_Id = quote(Id == "WNC1" & Season == "Summer")),
        list(start = "2015-06-10", end = "2015-06-16",
         filter.expr = quote(!(day(Datetime) %in% c(13,14))),
         only_Id = quote(Id == "WOL2" & Season == "Summer")),
        list(start = "2015-06-10", end = "2015-06-16",
         filter.expr = quote(!(day(Datetime) %in% c(12,13,14))),
         only_Id = quote(Id == "WRC5" & Season == "Summer")),
        list(start = "2015-06-19", end = "2015-06-24",
         filter.expr = quote(!(day(Datetime) %in% c(20,21,22))),
         only_Id = quote(Id == "BLD3" & Season == "Summer")),
        list(start = "2015-06-19", end = "2015-06-25",
         filter.expr = quote(!(day(Datetime) %in% c(24))),
         only_Id = quote(Id == "SIM1" & Season == "Summer"))
    )

#keep the full dataset for visualization later
LLdata_full <- LLdata

#filter the dates that are relevant for the analysis
LLdata <-
LLdata %>% filter_Datetime_multiple(arguments = filter_list,
                                    filter_function = filter_Date,
                                    full.day = TRUE)
```

#### Overview of the remaining days

Code

```
#create and save a figure overview of the remaining days
Figure.S2 <- 
LLdata %>% 
 gg_days(group = day(Datetime),
   geom = "ribbon", alpha = 0.25, fill = "#EFC000", col = "#EFC000", 
   linewidth = 0.25, x.axis.breaks = waiver(),
   x.axis.format = waiver(),
   y.axis.label = "melanopic EDI (lx)",
   x.axis.label = "Dates",
   x.axis.limits = \(x) Datetime_limits(x, length = ddays(7)))+ 
  facet_wrap(Id ~ Season, scales = "free_x", ncol = 2, strip.position = "left")+
  theme(axis.text=element_text(size=6),
        text = element_text(size=8),
        strip.text = element_text(size=6),
        panel.spacing = unit(0.1, "lines"))

Figure.S2
```

Code

```
ggsave("Figures/FigureS2.png", Figure.S2, height = 20, width = 17, units='cm')
```

#### Valid wear times

In the following section we will filter the data for valid wear times according to the `Status` column in the dataset. Unfortunately, because the data was stored in two different locale-settings, the column exists two times with slightly different names. The next section does necessary pre-processing to clean the data up.

Code

```
#clean up the data so that info that is stored in two columns is merged
LLdata <- LLdata %>% 
  mutate(Status = case_when(
    !is.na(Status..Nicht.am.Handgelenk.) ~ Status..Nicht.am.Handgelenk.,
    .default = NA_character_),
    Sleep = case_when(
    !is.na(Schlaf.Wach) ~ Schlaf.Wach,
    !is.na(S.W.Status) ~ S.W.Status,
    )) %>% 
  select(Id, Season, Datetime, MEDI, Status, Sleep, Aktivität)

#recode the data for Status and Sleep
LLdata <- LLdata %>% 
  mutate(Status = 
           case_when(Status == 0 ~ "non wear", Status == 1 ~ "wear") %>% 
           factor(levels = c("non wear", "wear")),
         Sleep = 
           case_when(Sleep == 0 ~ "sleep", Sleep == 1  ~ "wake") %>% 
           factor(levels = c("sleep", "wake")))
```

##### Non-Wear Times

Code

```
#create and save a figure that shows the non-wear times
Figure.S3 <- 
LLdata %>% 
 gg_days(group = interaction(day(Datetime), consecutive_id(Status)),
   geom = "ribbon", alpha = 0.25, aes_fill = Status, aes_col = Status, 
   linewidth = 0.25, x.axis.breaks = waiver(),
   x.axis.format = waiver(),
   y.axis.label = "melanopic EDI (lx)",
   x.axis.label = "Dates",
   x.axis.limits = \(x) Datetime_limits(x, length = ddays(7)))+ 
  facet_wrap(Id ~ Season, scales = "free_x", ncol = 2, strip.position = "left")+
    theme(axis.text=element_text(size=6),
        text = element_text(size=8),
        strip.text = element_text(size=6),
        panel.spacing = unit(0.1, "lines"),
        legend.position = "bottom",
        legend.margin=margin(0,0,0,0),
        legend.box.margin=margin(-10,-10,0,-10))

Figure.S3
```

Code

```
ggsave("Figures/FigureS3.png", Figure.S3, height = 20, width = 17, units='cm')
```

#### Included days

In this section we summarize the non-wear times by day and participant to judge whether a given participant-day has do be excluded from the analysis.

Code

```
#calculate the daily non-wear times as the percentage of non-wear times and
#check whether it exceeds the set threshold
Nonwear_summary <- 
LLdata %>% group_by(Id, Season, Day = date(Datetime)) %>% 
  summarize(NonWear = sum(Status == "non wear")/sum(!is.na(Status)),
            .groups = "drop_last") %>%
  mutate(valid_Day =
           (NonWear < (1-valid_data_threshold)) %>%
           factor() %>%
           forcats::fct_recode("Valid days" = "TRUE", "Invalid days" = "FALSE"),
         Id = forcats::fct_drop(Id)
         )

#create and save a figure that shows the non-wear times >0 by day and participant
Figure.2 <- 
Nonwear_summary %>% 
  filter(NonWear > 0) %>% 
  ggplot(aes(x= fct_reorder(interaction(Id, Day, sep = " "), NonWear), y = NonWear)) +
  geom_col() +
  geom_text(aes(label = vec_fmt_percent(NonWear)), 
            nudge_y = 0.02, size = 2.5, show.legend = FALSE) +
  labs(x = "Participant-Day", y = "Non-Wear Time", fill = "Participant") +
  coord_flip() +
  theme_minimal()+
  scale_y_continuous(labels = scales::label_percent(accuracy = 1))+
  guides(fill = "none")+
  gghighlight::gghighlight(
    NonWear > 1- valid_data_threshold, use_direct_label = FALSE)+
  geom_hline(yintercept = 1- valid_data_threshold, linetype = "dashed", 
             color = "red", linewidth = 0.25) +
  annotate(geom = "text", label = "Threshold criterion\nfor valid days", 
           x = 10, y = 1.005- valid_data_threshold, hjust = 0, vjust = 0, 
           color = "red", size = 3)+
    theme(axis.text=element_text(size=8))

Figure.2
```

Code

```
ggsave("Figures/Figure2.png", Figure.2, height = 8.5, width = 13, units='cm')

#summarize the non-wear times as a table
Nonwear_summary %>% 
  gtsummary::tbl_summary(
    statistic = list(NonWear ~"{min} - {max}"),
    label = list(NonWear ~ "Non-Wear Time",
                 valid_Day ~ "Valid Days"),
    by = valid_Day)
```

| **Characteristic** | **Valid days**, N = 881 | **Invalid days**, N = 41 |
| --- | --- | --- |
| Id |  |  |
| AET3 | 5 (5.7%) | 0 (0%) |
| BLD3 | 8 (9.1%) | 0 (0%) |
| CET8 | 7 (8.0%) | 0 (0%) |
| D7EB | 8 (9.1%) | 0 (0%) |
| DWM1 | 8 (9.1%) | 0 (0%) |
| HNN1 | 7 (8.0%) | 0 (0%) |
| LIR1 | 4 (4.5%) | 0 (0%) |
| MES8 | 7 (8.0%) | 0 (0%) |
| MIH2 | 6 (6.8%) | 0 (0%) |
| SIM1 | 8 (9.1%) | 0 (0%) |
| WNC1 | 5 (5.7%) | 0 (0%) |
| WOL2 | 7 (8.0%) | 3 (75%) |
| WRC5 | 8 (9.1%) | 1 (25%) |
| Season |  |  |
| Winter | 46 (52%) | 3 (75%) |
| Summer | 42 (48%) | 1 (25%) |
| Day | 2015-01-14 to 2015-06-25 | 2015-01-14 to 2015-06-16 |
| Non-Wear Time | 0.00 - 0.15 | 0.24 - 0.34 |
|  |  |  |
| --- | --- | --- |
| 1 n (%); Range | | |

Code

```
#connect valid and non-valid days to the data
LLdata <- 
  LLdata %>% group_by(Id, Season, Day = date(Datetime)) %>% left_join(Nonwear_summary) 

LLdata_full <- 
  LLdata_full %>% group_by(Id, Season, Day = date(Datetime)) %>% 
  left_join(Nonwear_summary) %>% 
  filter_Date(start = "2015-06-10", end = "2015-06-16",
              only_Id = Id == "WOL2" & Season == "Summer")

#create and save a figure about the illuminance on valid, invalid and non-dayshift days
Figure.3 <- 
LLdata_full %>% 
 gg_days(group = day(Datetime),
   geom = "ribbon", alpha = 0.25, aes_fill = valid_Day, aes_col = valid_Day,
   linewidth = 0.1, x.axis.breaks = waiver(),
   x.axis.format = waiver(),
   y.axis.label = "melanopic EDI (lx)",
   x.axis.label = "Dates",
   x.axis.limits = \(x) Datetime_limits(x, length = ddays(7)))+ 
  facet_wrap(Id ~ Season, scales = "free_x", ncol = 2, strip.position = "left")+
  scale_fill_discrete(type = c("#EFC000", "red"), 
                      labels = c("Valid", "Invalid", "non Dayshift (excluded)"))+
  scale_color_discrete(type = c("#EFC000", "red"), 
                       labels = c("Valid", "Invalid", "non Dayshift (excluded)"))+
    theme(axis.text=element_text(size=5.5),
        text = element_text(size=8),
        strip.text = element_text(size=6),
        panel.spacing = unit(0.1, "lines"),
        legend.position = "bottom",
        legend.margin=margin(0,0,0,0),
        legend.box.margin=margin(-10,-10,0,-10),
          axis.line = element_line(linewidth = 0.25),
          axis.ticks = element_line(linewidth = 0.25),
        legend.text = element_text(size=6),
        legend.title = element_text(size=7))+
  labs(fill = "Inclusion", color = "Inclusion")

Figure.3
```

Code

```
ggsave("Figures/Figure3.png", Figure.3, height = 21, width = 17, units='cm')

#filter the data to only include valid days
LLdata <- LLdata %>% 
  filter(valid_Day == "Valid days") %>% 
  select(-valid_Day, -NonWear)
```

Code

```
#create and save a figure about the illuminance between season
Figure.S4 <- 
LLdata %>% 
  ggplot(aes(x = MEDI, fill = Season)) + 
  geom_density_ridges(aes(y = Id, height = ..density..), stat = "density", alpha = 0.5) + 
  geom_vline(xintercept = 250, col = "red", linetype = "dashed") +
  scale_x_continuous(
    breaks = c(0, 1, 10, 100, 250, 1000, 10000),
    trans = "symlog",
    labels = \(x) format(x, scientific = FALSE, big.mark = " ")) + 
  theme_cowplot()+
  labs(x = "melanopic EDI (lux)", y = "Participant ID")+
  theme(legend.position = "bottom")

Figure.S4
```

Code

```
ggsave("Figures/FigureS4.png", Figure.S4, height = 17, width = 17, units='cm')
```

### Metrics

In this section we calculate the various metrics for the analysis. The following metrics2 have been selected, all based on melanopic EDI:

- Geometric mean and standard deviation
- Luminous exposure (lx\*h)
- Time above 250 lux (h)
- Time above 1000 lux (h)
- Mean timing of light above 250 lux (h)
- Mean timing of light below 10 lux (h)
- Intradaily variability
- Mean across the darkest (L5) and brightest (M10) hours
- Midpoint of the darkest (L5) and brightest (M10) hours

Code

```
#calculate the metrics
Metrics_dataset <- 
  LLdata %>% 
  summarize(
    across(
      .cols = MEDI,
      .fns = list(
        `Geometric mean (lx)` = \(x) geomean(x, na.rm = TRUE),
        `Geometric sd (lx)` = \(x) geosd(x, na.rm = TRUE),
        `Luminous exposure (lx*h)` = \(x) sum(x, na.rm = TRUE)*30/60/60,
        `Time above 250 lx (h)` = \(x) tat(x, 250, 30, "hours", as_df = FALSE),
        `Time above 1000 lx (h)` = 
          \(x) tat(x, 1000, 30, "hours", as_df = FALSE),
        `Mean timing of light above 250 lx (hh:mm)` = 
          \(x) {mlit(x, Datetime, 250, as_df = FALSE) %>% 
          as_datetime() %>% hms::as_hms()},
        `Mean timing of light below 10 lx (hh:mm)` = 
          \(x) {mlit(x, Datetime, 10, as_df = FALSE) %>% 
          as_datetime() %>% hms::as_hms()},
        `Intradaily variability` = 
          \(x) intradaily_variability(x, Datetime, as_df = FALSE),
        `Mean of darkest 5 hours (L5, lux)` = 
          \(x) bright_dark_period(
            x, Datetime, "dark", "5 hours", 30, loop = TRUE, as_df = FALSE
            ) %>% .[1],
        `Midpoint of darkest 5 hours (L5, hh:mm)` = 
          \(x) bright_dark_period(
            x, Datetime, "dark", "5 hours", 30, loop = TRUE, as_df = FALSE
            ) %>% .[2] %>% 
          as_datetime() %>% hms::as_hms(),
        `Mean of brightest 10 hours (M10, lux)` = 
          \(x) bright_dark_period(
            x, Datetime, "bright", "10 hours", 30, loop = TRUE, as_df = FALSE
            ) %>% .[1],
        `Midpoint of brightest 10 hours (M10, hh:mm)` = 
          \(x) bright_dark_period(
            x, Datetime, "bright", "10 hours", 30, loop = TRUE, as_df = FALSE
            ) %>% .[2] %>% 
          as_datetime() %>% hms::as_hms()
        ),
      .names = "{.fn}"),
    .groups = "drop_last"
  )

#styling formula for time
style_time <- function(x, format = "%H:%M"){
  x %>% hms::as_hms() %>% as.POSIXlt() %>% format(format)
}

#create a table-summary of the metrics
Metrics_dataset %>% 
  ungroup() %>% 
  select(-Day, -Id) %>% 
  tbl_summary(by = Season, missing_text = "Days without Metric",
              digits = list(
                `Midpoint of darkest 5 hours (L5, hh:mm)` ~ style_time,
                `Midpoint of brightest 10 hours (M10, hh:mm)` ~ style_time,
                `Mean timing of light above 250 lx (hh:mm)` ~ style_time,
                `Mean timing of light below 10 lx (hh:mm)` ~ style_time
                )
  )
```

| **Characteristic** | **Winter**, N = 461 | **Summer**, N = 421 |
| --- | --- | --- |
| Geometric mean (lx) | 1.23 (0.65, 1.94) | 3.39 (2.31, 5.90) |
| Geometric sd (lx) | 26 (14, 135) | 33 (21, 121) |
| Luminous exposure (lx\*h) | 705 (462, 1,091) | 4,052 (2,429, 6,881) |
| Time above 250 lx (h) | 0.56 (0.33, 0.90) | 2.34 (1.66, 3.37) |
| Time above 1000 lx (h) | 0.03 (0.00, 0.08) | 0.75 (0.47, 1.21) |
| Mean timing of light above 250 lx (hh:mm) | 13:31 (11:58, 14:20) | 13:37 (12:41, 14:40) |
| Days without Metric | 1 | 0 |
| Mean timing of light below 10 lx (hh:mm) | 11:40 (11:08, 12:17) | 12:37 (11:56, 13:10) |
| Intradaily variability | 0.84 (0.63, 1.37) | 1.23 (0.95, 1.60) |
| Mean of darkest 5 hours (L5, lux) | 0.05 (0.01, 0.12) | 0.06 (0.03, 0.14) |
| Midpoint of darkest 5 hours (L5, hh:mm) | 01:57 (01:18, 02:29) | 02:11 (01:28, 02:29) |
| Mean of brightest 10 hours (M10, lux) | 69 (43, 104) | 380 (224, 655) |
| Midpoint of brightest 10 hours (M10, hh:mm) | 11:19 (10:54, 11:44) | 12:52 (11:34, 13:55) |
|  |  |  |
| --- | --- | --- |
| 1 Median (IQR) | | |

### Bootstrapping

In this section we implement and perform the bootstrapping procedure to obtain the power estimates depending on sample size. We implement the following mixed-effect formula for our model:

`Metric ~ Season + (1|Id)`

Code

```
#change the hms columns to numeric
Metrics_dataset <- 
  Metrics_dataset %>% mutate(across(where(hms::is_hms), as.numeric))

#pivot data so that we have one row per metric
Metrics_dataset2 <- 
Metrics_dataset %>% 
  pivot_longer(c(-Id, -Season, -Day), 
               names_to = "Name", values_to = "Metric")

#function definition to perform the bootstrapping
resample_within_id <- 
  function(data, 
           n_participants,
           n_replicates = 2,
           seed = NULL){
    #set a seed
    if(!is.null(seed)) set.seed(seed)
    
    #prepare the data
    data <- data %>% ungroup() %>% nest(data = -Id)
    
    #create a dataframe with the resamples
    participants <- 
      1:n_replicates %>% 
      map(\(x)
       data %>% 
         sample_n(n_participants, replace = TRUE)
       ) %>% 
      list_rbind(names_to = "resamples") %>% 
      mutate(Id = paste0(Id, "_", 1:n_participants) %>% 
               factor(levels = unique(.))
             )
    
    #un- and re-nest the data, with resampling of days on a season/participant/metric level
    participants %>% unnest(data) %>%
      group_by(resamples, Season, Id, Name) %>%
      sample_frac(replace = TRUE) %>%
      ungroup() %>% nest(data = c(-resamples, -Name)) %>%
      mutate(sample_size = n_participants, .before = 1)
  }

#perfom the bootstrapping
resampled_data <-
  sample_size %>%
  map(\(x)
      {resample_within_id(Metrics_dataset2, n_participants = x, n_samples)}
    ) %>% list_rbind()
```

Code

```
#create the formula
Formula <- Metric ~ Season + (1|Id)

#perform the statistical analysis
boostrappedModels <- 
resampled_data %>% 
  rowwise() %>%  
  mutate(model = list(lmerTest::lmer(Formula, data = data)),
         pvalue = model %>% lmerTest::difflsmeans() %>% .$`Pr(>|t|)`) %>% 
  select(sample_size, Name, pvalue)

#compare the significance level of the test with the threshold significance
#calculate power based on the fraction of significant results (mean of trues)
#nest the data for plotting
Power_data <- 
boostrappedModels %>% 
  mutate(sign = pvalue <= sign_level) %>% 
  group_by(Name, sample_size) %>% 
  summarize(
    power = mean(sign),
    .groups = "keep"
  ) %>% 
  nest(data = -Name)

#save the power data
write_csv(Power_data %>% unnest(cols = data), "Results/Power_data.csv")

#if needed, load a previously calculated power data
# Power_data <- read_csv("Results/Power_data.csv") %>% nest(data = -Name)
```

### Results

In this section we summarize the results of the bootstrapping procedure.

Code

```
#create a plot function that takes the Power_data for one metric and plots it
Plot_function <- function(data, name) {
  data %>% 
    ggplot(aes(x = sample_size, y = power)) + geom_col() + 
  gghighlight(power >= Power_level, use_direct_label = FALSE) +
  geom_hline(yintercept = Power_level, col = "red", linetype = "dashed",
             linewidth = 0.25) + 
  theme_cowplot() + 
  annotate("label", x = mean(sample_size), y = Power_level+0.1, alpha = 0.75,
           label = "Required Power level", col = "red", size = 1.5,
           label.size = 0.2) + 
  labs(x = "Sample size", y = "Power") +
  scale_x_continuous(breaks = c(3,5, seq(10, max(sample_size), by = 10)))+
    scale_y_continuous(labels = scales::percent_format(accuracy = 1))+
  coord_cartesian(expand = FALSE, ylim = c(0,1))+
    ggtitle(name)+
    theme(plot.title = element_text(size = 5.5),
          axis.text= element_text(size=6),
          axis.title= element_text(size=6),
          axis.line = element_line(linewidth = 0.25),
          axis.ticks = element_line(linewidth = 0.25)
          )
}

#apply all metrics to the plot function
Power_data <- 
  Power_data %>% 
  mutate(plot = map2(data, Name, Plot_function))

#create all plots in a grid and save it
Figure.4 <- 
  Power_data$plot %>% 
  reduce(\(x, y) x + y) + 
  plot_annotation(tag_levels = 'A') & 
  theme(plot.tag = element_text(size = 8))
Figure.4
```

Code

```
ggsave("Figures/Figure4.png", Figure.4, height = 15, width = 22.5, units='cm')
```

Code

```
#get the required sample size for each metric
Power_summary <- 
Power_data %>% select(-plot) %>% unnest(data) %>% 
  mutate(Power_reached = power >= Power_level) %>% 
  filter(Power_reached, .preserve = TRUE) %>% 
  slice_min(sample_size) %>% 
  select(-Power_reached) %>% 
  ungroup() 

#check which metrics did not reach the threshold
set1 <- Power_summary$Name
set2 <- Power_data$Name
missing <- base::setdiff(set2, set1)


Power_summary %>% 
  arrange(sample_size, power) %>%
  gt() %>% 
  gt::cols_label(
    sample_size = "required Sample Size",
    power = "Power",
    Name = "Metric"
  ) %>% 
  fmt_percent(columns = power, decimals = 0) %>% 
  tab_footnote(footnote = "Power at the required sample size", 
               locations = gt::cells_column_labels(columns = power),
               ) %>% 
    tab_footnote(
    footnote = md(paste0("Metrics that did not reach the threshold: **", 
                      missing %>% paste0(collapse = ", "), "**")),
  ) %>% 
    tab_footnote(
    footnote = 
      paste0(
        "The sample size calculation is based on a bootstrap resampling of daily metrics between winter and summer seasons for ",
        N_participants,
        " participants. For each resampled dataset, significance was tested in a mixed-effect model with a significance level of ", 
        sign_level, 
        ". The fraction of significant differences was compared against the power level threshold of ", 
        Power_level, 
        ". The required sample size is the minimum sample size that reaches this threshold, with ", 
        n_samples, 
        " resamples per sample size (sample sizes from ", 
        min(sample_size), 
        " to ", 
        max(sample_size), 
        " were tested). The total amount of resamples/tests is ", 
        nrow(resampled_data), 
        " across all metrics."),
    locations = gt::cells_column_labels(columns = sample_size)
  )
```

| Metric | required Sample Size1 | Power2 |
| --- | --- | --- |
| Midpoint of brightest 10 hours (M10, hh:mm) | 3 | 81% |
| Luminous exposure (lx\*h) | 3 | 98% |
| Time above 1000 lx (h) | 3 | 98% |
| Geometric mean (lx) | 3 | 98% |
| Mean of brightest 10 hours (M10, lux) | 3 | 98% |
| Time above 250 lx (h) | 3 | 100% |
| Mean timing of light below 10 lx (hh:mm) | 5 | 81% |
| Intradaily variability | 17 | 80% |
| Mean of darkest 5 hours (L5, lux) | 24 | 81% |
| Mean timing of light above 250 lx (hh:mm) | 45 | 80% |
|  |  |  |
| --- | --- | --- |
| Metrics that did not reach the threshold: **Geometric sd (lx), Midpoint of darkest 5 hours (L5, hh:mm)** | | |
| 1 The sample size calculation is based on a bootstrap resampling of daily metrics between winter and summer seasons for 13 participants. For each resampled dataset, significance was tested in a mixed-effect model with a significance level of 0.05. The fraction of significant differences was compared against the power level threshold of 0.8. The required sample size is the minimum sample size that reaches this threshold, with 1000 resamples per sample size (sample sizes from 3 to 50 were tested). The total amount of resamples/tests is 576000 across all metrics. | | |
| 2 Power at the required sample size | | |

## Footnotes

1. Price, L. L. A., Khazova, M., & Udovicic, L. (2021). Assessment of the Light Exposures of Shift-working Nurses in London and Dortmund in Relation to Recommendations for Sleep and Circadian Health. Ann Work Expo Health. https://doi.org/10.1093/annweh/wxab092↩︎
2. More Information about those metrics can be found under: Hartmeyer S, Andersen M. Towards a framework for light-dosimetry studies: Quantification metrics. Lighting Research & Technology. 2023;0(0). doi:10.1177/14771535231170500↩︎


##### Source Code

```
---
title: "Power analysis for personal light exposure measurements and interventions"
subtitle: "Supplementary Document S1 - Statistical Analysis"
author: "Johannes Zauner, Ljiljana Udovicic, Manuel Spitschan"
format: 
  html:
    code-fold: true
    embed-resources: true
    code-tools: true
    toc: true
params:
  Power_level: 0.8
  sign_level: 0.05
  valid_data_threshold: 0.8
  n_samples: 1000
  sample_size_min: 3
  sample_size_max: 50
  seed: 20231212
---

## Preface

This is a supplementary document to the main manuscript of the same title. The following statistical analysis evaluates the necessary sample size to significantly detect a difference in metrics derived from wearable light logger data. The data were provided by [BAuA](https://www.baua.de/DE/Home/Home_node.html) and were collected as part of a study[^1] on evaluating personal light exposure between London and Dortmund.

[^1]: Price, L. L. A., Khazova, M., & Udovicic, L. (2021). Assessment of the Light Exposures of Shift-working Nurses in London and Dortmund in Relation to Recommendations for Sleep and Circadian Health. Ann Work Expo Health. https://doi.org/10.1093/annweh/wxab092

The goal of this analysis is to provide a sensible sample size for a field test study of similar design. Data analysis will be performed in `R` statistical software, using the [`LightLogR`](https://tscnlab.github.io/LightLogR/) R package, which is part of the [MeLiDos](www.melidos.eu) project.

```{r}
#| output: false

#collect all the packages that are needed for the analysis
packages <- c("quarto", "tidyverse", "broom", "cowplot", "gt", "gtsummary", 
              "gghighlight", "patchwork", "readxl", "lme4", "lmerTest",
              "ggridges", "osfr")
packages_github <- c("tscnlab/LightLogR", "steffenhartmeyer/lightdosimetry")

#check if packages are installed, if not install them
if(!require(pacman)) {
  install.packages("pacman") 
  library(pacman)
  }

p_load(packages, character.only = TRUE)
p_load_gh(char = packages_github)

#at the time of writing, lme4 might need to be reinstalled from source, due to
#an update of the Matrix package (https://stat.ethz.ch/pipermail/r-package-devel/2023q4/010054.html)
# oo <- options(repos = "https://cran.r-project.org/")
# install.packages("Matrix", type = "source")
# install.packages("lme4", type = "source")
# options(oo)
# library(lme4)
# library(lmerTest)

#major parameters for the analysis
Power_level <- params$Power_level
sign_level <- params$sign_level
valid_data_threshold <- params$valid_data_threshold
n_samples <-  params$n_samples
sample_size <- params$sample_size_min:params$sample_size_max

```

### Analysis Outline

The analysis will calculate the necessary sample size to reach a power level of `r Power_level` for a range of relevant metrics of personal light exposure. These metrics will be included in `LightLogR` by the time the manuscript is published, but are presently taken from the [`lightdosimetry`](https://github.com/steffenhartmeyer/lightdosimetry/) package by Steffen Hartmeyer.

#### General info

To be included in the analysis, each participant-day needs to have a minimum of `r vec_fmt_percent(valid_data_threshold)` valid, non-missing data. Non-missing data is evaluated by a regular sequence of measurement intervals (epochs) that are dominant to the data. Valid data is evaluated by a column of the data that indicates whether the device was worn or not.

The various metrics are calculated for each participant-day. This dataset will be the basis for the analysis.

#### Analysis

The analysis uses a `bootstrap` method, i.e., it resamples the data with replacement to generate a distribution of the metric of interest (`r n_samples` resamples). The distribution is generated for both the January and June data, and within participant. Each resampled dataset is tested with a `linear mixed-effect` model to test for a significant difference between the two seasons, within participants, for each given metric. The percentage of resamples that are significant are counted and used to calculate the power level.

The analysis is repeated for a range of sample sizes (min = `r min(sample_size)`, max = `r max(sample_size)`), and the sample size that reaches the required `Power Level` is recorded and reported.

## Import and Data Preparation

### Seed
Here we set a seed for the random number generator, to ensure reproducibility of the analysis.

```{r Seed}
#set a seed for reproducibility
set.seed(params$seed)
```

### Import

This section will import all the data and provide an overview as well as first measures to check and secure the data quality.
A total of 26 files are in the folder (13 participants, 2 seasons each). They are, however, not consistent in their encoding, so we need to import them separately and then combine them. Also, two of them are kept in excel-files, which also requires separate handling.

```{r Import}

#list all the Participant Id's
Participant.IDs <- c("WRC5", "WOL2", "WNC1", "SIM1", "MIH2", "MES8", "LIR1",
                     "HNN1", "DWM1", "D7EB", "CET8", "BLD3", "AET3")

N_participants <- length(Participant.IDs)

#list all the files
files <- list.files(path = "Data", pattern = "*.csv", full.names = TRUE)
files_xlsx <- list.files(path = "Data", pattern = "*.xlsx", full.names = TRUE)

#extract only files for the participants in the list, using stringr
files <- files[str_detect(files, str_c(Participant.IDs, collapse = "|"))]
files_xlsx <- files_xlsx[str_detect(files_xlsx, str_c(Participant.IDs, collapse = "|"))]

#general import settings for the data, as it is a german dataset
column_names <- c("Zeile", "Datum", "Zeit", "Status") #this is a sequence of column 
german_encoding <- c(2, 4, 6, 8, 11, 13, 14, 16, 22) #these are the files that use german encoding
int_encoding <- base::setdiff(1:24, german_encoding) #these are the files that use standard encoding
na_s <- c("", "NA", "kZ") #these are the NA strings in the files
auto.id <- "^\\d{5}_(.{4})" #this is the regex to extract the participant id
tz <- "Europe/Berlin" #this is the timezone of the data

#read in the files with german encoding
LLdata <- import$Actiwatch_Spectrum(files[german_encoding], 
                                    locale = locale(encoding="latin1"),
                                    tz = tz, na = na_s, auto.id = auto.id,
                                    column_names = column_names)

#read in the files with english encoding
LLdata2 <- import$Actiwatch_Spectrum(files[int_encoding], 
                                    tz = tz, na = na_s, auto.id = auto.id,
                                    column_names = column_names
                                    )

#read all excel files in and clean them up to be in comparable shape
LLdata3 <- 
  read_xlsx(
    files_xlsx[1], skip =  149, na = na_s, .name_repair = "universal",
    )[-1, ]
LLdata4 <- 
  read_xlsx(
    files_xlsx[2], skip = 164, na = na_s, .name_repair = "universal"
    )[-1, ]

LLdata_xlsx <- 
  list(LLdata3, LLdata4) %>% 
  map2(files_xlsx,
       \(file, filenames) {
         file %>% 
           mutate(file.name = 
                    filenames %>% basename() %>% tools::file_path_sans_ext(),
                  Id = str_extract(file.name, auto.id, group = 1),
                  Zeit = hms::as_hms(Zeit),
                  Datetime = as.POSIXct(paste(Datum, Zeit), 
                                        format = "%Y-%m-%d %H:%M:%S", tz = "UTC") %>% 
                    force_tz(tz),
                  Zeile = as.integer(Zeile),
                  across(
                    c(Status..Nicht.am.Handgelenk., Markierung, Schlaf.Wach, 
                      Intervallstatus), as.factor))
       }) %>% 
  list_rbind()

#combine the two datasets
LLdata <- join_datasets(LLdata, LLdata2, LLdata_xlsx)
rm(LLdata2, LLdata_xlsx, LLdata3, LLdata4)

#differentiate the datasets by Season
LLdata <- 
  LLdata %>%
  group_by(Id, Season = quarter(Datetime)) %>% 
  mutate(
    Season = case_when(Season == 1 ~ "Winter", Season == 2 ~ "Summer") %>% 
      factor(levels = c("Winter", "Summer")))

#get a general overview
Figure.1 <- 
  LLdata %>% gg_overview() + 
  labs(x = "Month", y = "Participant ID") +
  scale_x_datetime(date_breaks = "1 month", date_labels = "%b") +
  coord_cartesian(xlim = c(as.POSIXct("2015-01-09", tz = tz), NA)) +
  theme(axis.text=element_text(size=8),
        text = element_text(size = 10),
        axis.line = element_line(linewidth = 0.25),
        axis.ticks = element_line(linewidth = 0.25))
Figure.1

#save the figure
ggsave("Figures/Figure1.png", Figure.1, height = 8.5, width = 8.5, units='cm')

```

The import doesn't suggest that there are any gaps in the dataset, but just to make sure, let us search for gaps in the data with the new grouping.

```{r gaps}
#| code-fold: show
#search for gaps (none are found)
LLdata %>% gap_finder()
```

Now lets go into a more comprehensive overview.

```{r overview}
#| fig-height: 20
#| fig-width: 13

#create and save a figure with an overview of the illuminance values
Figure.S1 <- 
LLdata %>% 
 gg_days(y.axis = Weißes.Licht, x.axis.breaks = waiver(), linewidth = 0.1,
   x.axis.format = waiver(), 
   y.axis.label = "Illuminance (lx)", x.axis.label = "Dates")+ 
  facet_wrap(Id ~ Season, scales = "free_x", ncol = 2, strip.position = "left") +
  theme(axis.text=element_text(size=6),
        text = element_text(size=8),
        strip.text = element_text(size=6),
        panel.spacing = unit(0.1, "lines"))

Figure.S1
ggsave("Figures/FigureS1.png", Figure.S1, height = 20, width = 17, units='cm')


```

We can see some variation in the data, but nothing unexpected.

### Preparing the Data

There are several steps required before we can go into metrics calculation:

-   We need to calculate the `melanopic EDI` (MEDI), as these are not part of the dataset. `BAuA` has provided a calibration function that is based on their measurements of the device.
-   We need to filter for dates that are relevant for the analysis, i.e., that are `day-shifts`.

#### Calculating melanopic EDI

```{r MEDI}
#calculate melanopic EDI based on the BAuA calibration
LLdata <- LLdata %>% mutate(MEDI = (Grünes.Licht+ Blaues.Licht)*4.3/1.3262)
```

#### Filtering for Day-Shifts

Sadly, the days with day-shift is not part of the dataset, but there is a manually collected list with that information. The `filter_list` will collect these dates and we filter the data accordingly with the `filter_Datetime_multiple()` function.

```{r filtering}
#gathering the dates for the filter
filter_list <- 
  list(
    #Winter
    #Consecutive Days
    list(start = "2015-01-14", end = "2015-01-16", 
         only_Id = quote(Id == "AET3" & Season == "Winter")),
    list(start = "2015-01-19", end = "2015-01-20", 
         only_Id = quote(Id == "HNN1" & Season == "Winter")),
    list(start = "2015-01-16", end = "2015-01-17", 
         only_Id = quote(Id == "LIR1" & Season == "Winter")),
    list(start = "2015-01-23", end = "2015-01-25", 
         only_Id = quote(Id == "MIH2" & Season == "Winter")),
    list(start = "2015-01-26", end = "2015-01-27", 
         only_Id = quote(Id == "WNC1" & Season == "Winter")),
    list(start = "2015-01-14", end = "2015-01-18",
         only_Id = quote(Id == "WRC5" & Season == "Winter")),
    list(start = "2015-01-28", end = "2015-01-29",
         only_Id = quote(Id == "SIM1" & Season == "Winter")),
    #Days with gaps
    list(start = "2015-01-14", end = "2015-01-20",
         filter.expr = quote(!(day(Datetime) %in% c(17, 18))),
         only_Id = quote(Id == "CET8" & Season == "Winter")),
    list(start = "2015-01-14", end = "2015-01-20",
         filter.expr = quote(!(day(Datetime) %in% c(17, 18))),
         only_Id = quote(Id == "DWM1" & Season == "Winter")),
    list(start = "2015-01-14", end = "2015-01-20",
         filter.expr = quote(!(day(Datetime) %in% c(17, 18))),
         only_Id = quote(Id == "D7EB" & Season == "Winter")),
    list(start = "2015-01-14", end = "2015-01-20",
         filter.expr = quote(!(day(Datetime) %in% c(17, 18))),
         only_Id = quote(Id == "MES8" & Season == "Winter")),
    list(start = "2015-01-23", end = "2015-01-29",
         filter.expr = quote(!(day(Datetime) %in% c(24, 25))),
         only_Id = quote(Id == "BLD3" & Season == "Winter")),
    list(start = "2015-01-14", end = "2015-01-20",
         filter.expr = quote(!(day(Datetime) %in% c(17, 18))),
         only_Id = quote(Id == "WOL2" & Season == "Winter")),
    #Summer
    #Consecutive Days    
    list(start = "2015-06-15", end = "2015-06-16", 
         only_Id = quote(Id == "AET3" & Season == "Summer")),
    list(start = "2015-06-11", end = "2015-06-12", 
         only_Id = quote(Id == "CET8" & Season == "Summer")),
    list(start = "2015-06-20", end = "2015-06-22", 
         only_Id = quote(Id == "DWM1" & Season == "Summer")),
    list(start = "2015-06-23", end = "2015-06-25", 
         only_Id = quote(Id == "D7EB" & Season == "Summer")),
    list(start = "2015-06-10", end = "2015-06-14", 
         only_Id = quote(Id == "HNN1" & Season == "Summer")),
    list(start = "2015-06-15", end = "2015-06-16", 
         only_Id = quote(Id == "LIR1" & Season == "Summer")),
    list(start = "2015-06-13", end = "2015-06-14", 
         only_Id = quote(Id == "MES8" & Season == "Summer")),
    list(start = "2015-06-10", end = "2015-06-12", 
         only_Id = quote(Id == "MIH2" & Season == "Summer")),
    #Days with gaps
        list(start = "2015-06-20", end = "2015-06-25",
         filter.expr = quote(!(day(Datetime) %in% c(21,22,23))),
         only_Id = quote(Id == "WNC1" & Season == "Summer")),
        list(start = "2015-06-10", end = "2015-06-16",
         filter.expr = quote(!(day(Datetime) %in% c(13,14))),
         only_Id = quote(Id == "WOL2" & Season == "Summer")),
        list(start = "2015-06-10", end = "2015-06-16",
         filter.expr = quote(!(day(Datetime) %in% c(12,13,14))),
         only_Id = quote(Id == "WRC5" & Season == "Summer")),
        list(start = "2015-06-19", end = "2015-06-24",
         filter.expr = quote(!(day(Datetime) %in% c(20,21,22))),
         only_Id = quote(Id == "BLD3" & Season == "Summer")),
        list(start = "2015-06-19", end = "2015-06-25",
         filter.expr = quote(!(day(Datetime) %in% c(24))),
         only_Id = quote(Id == "SIM1" & Season == "Summer"))
    )

#keep the full dataset for visualization later
LLdata_full <- LLdata

#filter the dates that are relevant for the analysis
LLdata <-
LLdata %>% filter_Datetime_multiple(arguments = filter_list,
                                    filter_function = filter_Date,
                                    full.day = TRUE)

```

#### Overview of the remaining days

```{r remaining days}
#| fig-height: 20
#| fig-width: 13
#| warning: FALSE

#create and save a figure overview of the remaining days
Figure.S2 <- 
LLdata %>% 
 gg_days(group = day(Datetime),
   geom = "ribbon", alpha = 0.25, fill = "#EFC000", col = "#EFC000", 
   linewidth = 0.25, x.axis.breaks = waiver(),
   x.axis.format = waiver(),
   y.axis.label = "melanopic EDI (lx)",
   x.axis.label = "Dates",
   x.axis.limits = \(x) Datetime_limits(x, length = ddays(7)))+ 
  facet_wrap(Id ~ Season, scales = "free_x", ncol = 2, strip.position = "left")+
  theme(axis.text=element_text(size=6),
        text = element_text(size=8),
        strip.text = element_text(size=6),
        panel.spacing = unit(0.1, "lines"))

Figure.S2
ggsave("Figures/FigureS2.png", Figure.S2, height = 20, width = 17, units='cm')


```

#### Valid wear times

In the following section we will filter the data for valid wear times according to the `Status` column in the dataset. Unfortunately, because the data was stored in two different locale-settings, the column exists two times with slightly different names. The next section does necessary pre-processing to clean the data up.

```{r cleanup}
#clean up the data so that info that is stored in two columns is merged
LLdata <- LLdata %>% 
  mutate(Status = case_when(
    !is.na(Status..Nicht.am.Handgelenk.) ~ Status..Nicht.am.Handgelenk.,
    .default = NA_character_),
    Sleep = case_when(
    !is.na(Schlaf.Wach) ~ Schlaf.Wach,
    !is.na(S.W.Status) ~ S.W.Status,
    )) %>% 
  select(Id, Season, Datetime, MEDI, Status, Sleep, Aktivität)

#recode the data for Status and Sleep
LLdata <- LLdata %>% 
  mutate(Status = 
           case_when(Status == 0 ~ "non wear", Status == 1 ~ "wear") %>% 
           factor(levels = c("non wear", "wear")),
         Sleep = 
           case_when(Sleep == 0 ~ "sleep", Sleep == 1  ~ "wake") %>% 
           factor(levels = c("sleep", "wake")))

```

##### Non-Wear Times

```{r nonwear times}
#| fig-height: 20
#| fig-width: 13
#| warning: FALSE

#create and save a figure that shows the non-wear times
Figure.S3 <- 
LLdata %>% 
 gg_days(group = interaction(day(Datetime), consecutive_id(Status)),
   geom = "ribbon", alpha = 0.25, aes_fill = Status, aes_col = Status, 
   linewidth = 0.25, x.axis.breaks = waiver(),
   x.axis.format = waiver(),
   y.axis.label = "melanopic EDI (lx)",
   x.axis.label = "Dates",
   x.axis.limits = \(x) Datetime_limits(x, length = ddays(7)))+ 
  facet_wrap(Id ~ Season, scales = "free_x", ncol = 2, strip.position = "left")+
    theme(axis.text=element_text(size=6),
        text = element_text(size=8),
        strip.text = element_text(size=6),
        panel.spacing = unit(0.1, "lines"),
        legend.position = "bottom",
        legend.margin=margin(0,0,0,0),
        legend.box.margin=margin(-10,-10,0,-10))

Figure.S3
ggsave("Figures/FigureS3.png", Figure.S3, height = 20, width = 17, units='cm')


```

#### Included days

In this section we summarize the non-wear times by day and participant to judge whether a given participant-day has do be excluded from the analysis.

```{r nonwear summary}
#| warning: FALSE

#calculate the daily non-wear times as the percentage of non-wear times and
#check whether it exceeds the set threshold
Nonwear_summary <- 
LLdata %>% group_by(Id, Season, Day = date(Datetime)) %>% 
  summarize(NonWear = sum(Status == "non wear")/sum(!is.na(Status)),
            .groups = "drop_last") %>%
  mutate(valid_Day =
           (NonWear < (1-valid_data_threshold)) %>%
           factor() %>%
           forcats::fct_recode("Valid days" = "TRUE", "Invalid days" = "FALSE"),
         Id = forcats::fct_drop(Id)
         )

#create and save a figure that shows the non-wear times >0 by day and participant
Figure.2 <- 
Nonwear_summary %>% 
  filter(NonWear > 0) %>% 
  ggplot(aes(x= fct_reorder(interaction(Id, Day, sep = " "), NonWear), y = NonWear)) +
  geom_col() +
  geom_text(aes(label = vec_fmt_percent(NonWear)), 
            nudge_y = 0.02, size = 2.5, show.legend = FALSE) +
  labs(x = "Participant-Day", y = "Non-Wear Time", fill = "Participant") +
  coord_flip() +
  theme_minimal()+
  scale_y_continuous(labels = scales::label_percent(accuracy = 1))+
  guides(fill = "none")+
  gghighlight::gghighlight(
    NonWear > 1- valid_data_threshold, use_direct_label = FALSE)+
  geom_hline(yintercept = 1- valid_data_threshold, linetype = "dashed", 
             color = "red", linewidth = 0.25) +
  annotate(geom = "text", label = "Threshold criterion\nfor valid days", 
           x = 10, y = 1.005- valid_data_threshold, hjust = 0, vjust = 0, 
           color = "red", size = 3)+
    theme(axis.text=element_text(size=8))

Figure.2
ggsave("Figures/Figure2.png", Figure.2, height = 8.5, width = 13, units='cm')

#summarize the non-wear times as a table
Nonwear_summary %>% 
  gtsummary::tbl_summary(
    statistic = list(NonWear ~"{min} - {max}"),
    label = list(NonWear ~ "Non-Wear Time",
                 valid_Day ~ "Valid Days"),
    by = valid_Day)

```

```{r valid days}
#| fig-height: 20
#| fig-width: 13
#| warning: FALSE

#connect valid and non-valid days to the data
LLdata <- 
  LLdata %>% group_by(Id, Season, Day = date(Datetime)) %>% left_join(Nonwear_summary) 

LLdata_full <- 
  LLdata_full %>% group_by(Id, Season, Day = date(Datetime)) %>% 
  left_join(Nonwear_summary) %>% 
  filter_Date(start = "2015-06-10", end = "2015-06-16",
              only_Id = Id == "WOL2" & Season == "Summer")

#create and save a figure about the illuminance on valid, invalid and non-dayshift days
Figure.3 <- 
LLdata_full %>% 
 gg_days(group = day(Datetime),
   geom = "ribbon", alpha = 0.25, aes_fill = valid_Day, aes_col = valid_Day,
   linewidth = 0.1, x.axis.breaks = waiver(),
   x.axis.format = waiver(),
   y.axis.label = "melanopic EDI (lx)",
   x.axis.label = "Dates",
   x.axis.limits = \(x) Datetime_limits(x, length = ddays(7)))+ 
  facet_wrap(Id ~ Season, scales = "free_x", ncol = 2, strip.position = "left")+
  scale_fill_discrete(type = c("#EFC000", "red"), 
                      labels = c("Valid", "Invalid", "non Dayshift (excluded)"))+
  scale_color_discrete(type = c("#EFC000", "red"), 
                       labels = c("Valid", "Invalid", "non Dayshift (excluded)"))+
    theme(axis.text=element_text(size=5.5),
        text = element_text(size=8),
        strip.text = element_text(size=6),
        panel.spacing = unit(0.1, "lines"),
        legend.position = "bottom",
        legend.margin=margin(0,0,0,0),
        legend.box.margin=margin(-10,-10,0,-10),
          axis.line = element_line(linewidth = 0.25),
          axis.ticks = element_line(linewidth = 0.25),
        legend.text = element_text(size=6),
        legend.title = element_text(size=7))+
  labs(fill = "Inclusion", color = "Inclusion")

Figure.3
ggsave("Figures/Figure3.png", Figure.3, height = 21, width = 17, units='cm')

#filter the data to only include valid days
LLdata <- LLdata %>% 
  filter(valid_Day == "Valid days") %>% 
  select(-valid_Day, -NonWear)

```

```{r illuminance season}
#| fig-height: 13
#| fig-width: 13
#| warning: FALSE

#create and save a figure about the illuminance between season
Figure.S4 <- 
LLdata %>% 
  ggplot(aes(x = MEDI, fill = Season)) + 
  geom_density_ridges(aes(y = Id, height = ..density..), stat = "density", alpha = 0.5) + 
  geom_vline(xintercept = 250, col = "red", linetype = "dashed") +
  scale_x_continuous(
    breaks = c(0, 1, 10, 100, 250, 1000, 10000),
    trans = "symlog",
    labels = \(x) format(x, scientific = FALSE, big.mark = " ")) + 
  theme_cowplot()+
  labs(x = "melanopic EDI (lux)", y = "Participant ID")+
  theme(legend.position = "bottom")

Figure.S4
ggsave("Figures/FigureS4.png", Figure.S4, height = 17, width = 17, units='cm')

```

### Metrics

In this section we calculate the various metrics for the analysis. The following metrics[^2] have been selected, all based on melanopic EDI:

[^2]: More Information about those metrics can be found under: Hartmeyer S, Andersen M. Towards a framework for light-dosimetry studies: Quantification metrics. Lighting Research & Technology. 2023;0(0). doi:10.1177/14771535231170500

-   Geometric mean and standard deviation
-   Luminous exposure (lx\*h)
-   Time above 250 lux (h)
-   Time above 1000 lux (h)
-   Mean timing of light above 250 lux (h)
-   Mean timing of light below 10 lux (h)
-   Intradaily variability
-   Mean across the darkest (L5) and brightest (M10) hours
-   Midpoint of the darkest (L5) and brightest (M10) hours

```{r metrics}
#| warning: FALSE

#calculate the metrics
Metrics_dataset <- 
  LLdata %>% 
  summarize(
    across(
      .cols = MEDI,
      .fns = list(
        `Geometric mean (lx)` = \(x) geomean(x, na.rm = TRUE),
        `Geometric sd (lx)` = \(x) geosd(x, na.rm = TRUE),
        `Luminous exposure (lx*h)` = \(x) sum(x, na.rm = TRUE)*30/60/60,
        `Time above 250 lx (h)` = \(x) tat(x, 250, 30, "hours", as_df = FALSE),
        `Time above 1000 lx (h)` = 
          \(x) tat(x, 1000, 30, "hours", as_df = FALSE),
        `Mean timing of light above 250 lx (hh:mm)` = 
          \(x) {mlit(x, Datetime, 250, as_df = FALSE) %>% 
          as_datetime() %>% hms::as_hms()},
        `Mean timing of light below 10 lx (hh:mm)` = 
          \(x) {mlit(x, Datetime, 10, as_df = FALSE) %>% 
          as_datetime() %>% hms::as_hms()},
        `Intradaily variability` = 
          \(x) intradaily_variability(x, Datetime, as_df = FALSE),
        `Mean of darkest 5 hours (L5, lux)` = 
          \(x) bright_dark_period(
            x, Datetime, "dark", "5 hours", 30, loop = TRUE, as_df = FALSE
            ) %>% .[1],
        `Midpoint of darkest 5 hours (L5, hh:mm)` = 
          \(x) bright_dark_period(
            x, Datetime, "dark", "5 hours", 30, loop = TRUE, as_df = FALSE
            ) %>% .[2] %>% 
          as_datetime() %>% hms::as_hms(),
        `Mean of brightest 10 hours (M10, lux)` = 
          \(x) bright_dark_period(
            x, Datetime, "bright", "10 hours", 30, loop = TRUE, as_df = FALSE
            ) %>% .[1],
        `Midpoint of brightest 10 hours (M10, hh:mm)` = 
          \(x) bright_dark_period(
            x, Datetime, "bright", "10 hours", 30, loop = TRUE, as_df = FALSE
            ) %>% .[2] %>% 
          as_datetime() %>% hms::as_hms()
        ),
      .names = "{.fn}"),
    .groups = "drop_last"
  )

#styling formula for time
style_time <- function(x, format = "%H:%M"){
  x %>% hms::as_hms() %>% as.POSIXlt() %>% format(format)
}

#create a table-summary of the metrics
Metrics_dataset %>% 
  ungroup() %>% 
  select(-Day, -Id) %>% 
  tbl_summary(by = Season, missing_text = "Days without Metric",
              digits = list(
                `Midpoint of darkest 5 hours (L5, hh:mm)` ~ style_time,
                `Midpoint of brightest 10 hours (M10, hh:mm)` ~ style_time,
                `Mean timing of light above 250 lx (hh:mm)` ~ style_time,
                `Mean timing of light below 10 lx (hh:mm)` ~ style_time
                )
  )

```

### Bootstrapping

In this section we implement and perform the bootstrapping procedure to obtain the power estimates depending on sample size. We implement the following mixed-effect formula for our model:

`Metric ~ Season + (1|Id)`

```{r bootstrapping}
#change the hms columns to numeric
Metrics_dataset <- 
  Metrics_dataset %>% mutate(across(where(hms::is_hms), as.numeric))

#pivot data so that we have one row per metric
Metrics_dataset2 <- 
Metrics_dataset %>% 
  pivot_longer(c(-Id, -Season, -Day), 
               names_to = "Name", values_to = "Metric")

#function definition to perform the bootstrapping
resample_within_id <- 
  function(data, 
           n_participants,
           n_replicates = 2,
           seed = NULL){
    #set a seed
    if(!is.null(seed)) set.seed(seed)
    
    #prepare the data
    data <- data %>% ungroup() %>% nest(data = -Id)
    
    #create a dataframe with the resamples
    participants <- 
      1:n_replicates %>% 
      map(\(x)
       data %>% 
         sample_n(n_participants, replace = TRUE)
       ) %>% 
      list_rbind(names_to = "resamples") %>% 
      mutate(Id = paste0(Id, "_", 1:n_participants) %>% 
               factor(levels = unique(.))
             )
    
    #un- and re-nest the data, with resampling of days on a season/participant/metric level
    participants %>% unnest(data) %>%
      group_by(resamples, Season, Id, Name) %>%
      sample_frac(replace = TRUE) %>%
      ungroup() %>% nest(data = c(-resamples, -Name)) %>%
      mutate(sample_size = n_participants, .before = 1)
  }

#perfom the bootstrapping
resampled_data <-
  sample_size %>%
  map(\(x)
      {resample_within_id(Metrics_dataset2, n_participants = x, n_samples)}
    ) %>% list_rbind()
```

```{r statistics}
#| warning: FALSE

#create the formula
Formula <- Metric ~ Season + (1|Id)

#perform the statistical analysis
boostrappedModels <- 
resampled_data %>% 
  rowwise() %>%  
  mutate(model = list(lmerTest::lmer(Formula, data = data)),
         pvalue = model %>% lmerTest::difflsmeans() %>% .$`Pr(>|t|)`) %>% 
  select(sample_size, Name, pvalue)

#compare the significance level of the test with the threshold significance
#calculate power based on the fraction of significant results (mean of trues)
#nest the data for plotting
Power_data <- 
boostrappedModels %>% 
  mutate(sign = pvalue <= sign_level) %>% 
  group_by(Name, sample_size) %>% 
  summarize(
    power = mean(sign),
    .groups = "keep"
  ) %>% 
  nest(data = -Name)

#save the power data
write_csv(Power_data %>% unnest(cols = data), "Results/Power_data.csv")

#if needed, load a previously calculated power data
# Power_data <- read_csv("Results/Power_data.csv") %>% nest(data = -Name)

```

### Results

In this section we summarize the results of the bootstrapping procedure.

```{r results plot}
#| fig-height: 13
#| fig-width: 15

#create a plot function that takes the Power_data for one metric and plots it
Plot_function <- function(data, name) {
  data %>% 
    ggplot(aes(x = sample_size, y = power)) + geom_col() + 
  gghighlight(power >= Power_level, use_direct_label = FALSE) +
  geom_hline(yintercept = Power_level, col = "red", linetype = "dashed",
             linewidth = 0.25) + 
  theme_cowplot() + 
  annotate("label", x = mean(sample_size), y = Power_level+0.1, alpha = 0.75,
           label = "Required Power level", col = "red", size = 1.5,
           label.size = 0.2) + 
  labs(x = "Sample size", y = "Power") +
  scale_x_continuous(breaks = c(3,5, seq(10, max(sample_size), by = 10)))+
    scale_y_continuous(labels = scales::percent_format(accuracy = 1))+
  coord_cartesian(expand = FALSE, ylim = c(0,1))+
    ggtitle(name)+
    theme(plot.title = element_text(size = 5.5),
          axis.text= element_text(size=6),
          axis.title= element_text(size=6),
          axis.line = element_line(linewidth = 0.25),
          axis.ticks = element_line(linewidth = 0.25)
          )
}

#apply all metrics to the plot function
Power_data <- 
  Power_data %>% 
  mutate(plot = map2(data, Name, Plot_function))

#create all plots in a grid and save it
Figure.4 <- 
  Power_data$plot %>% 
  reduce(\(x, y) x + y) + 
  plot_annotation(tag_levels = 'A') & 
  theme(plot.tag = element_text(size = 8))
Figure.4

ggsave("Figures/Figure4.png", Figure.4, height = 15, width = 22.5, units='cm')

```

```{r results table}

#get the required sample size for each metric
Power_summary <- 
Power_data %>% select(-plot) %>% unnest(data) %>% 
  mutate(Power_reached = power >= Power_level) %>% 
  filter(Power_reached, .preserve = TRUE) %>% 
  slice_min(sample_size) %>% 
  select(-Power_reached) %>% 
  ungroup() 

#check which metrics did not reach the threshold
set1 <- Power_summary$Name
set2 <- Power_data$Name
missing <- base::setdiff(set2, set1)


Power_summary %>% 
  arrange(sample_size, power) %>%
  gt() %>% 
  gt::cols_label(
    sample_size = "required Sample Size",
    power = "Power",
    Name = "Metric"
  ) %>% 
  fmt_percent(columns = power, decimals = 0) %>% 
  tab_footnote(footnote = "Power at the required sample size", 
               locations = gt::cells_column_labels(columns = power),
               ) %>% 
    tab_footnote(
    footnote = md(paste0("Metrics that did not reach the threshold: **", 
                      missing %>% paste0(collapse = ", "), "**")),
  ) %>% 
    tab_footnote(
    footnote = 
      paste0(
        "The sample size calculation is based on a bootstrap resampling of daily metrics between winter and summer seasons for ",
        N_participants,
        " participants. For each resampled dataset, significance was tested in a mixed-effect model with a significance level of ", 
        sign_level, 
        ". The fraction of significant differences was compared against the power level threshold of ", 
        Power_level, 
        ". The required sample size is the minimum sample size that reaches this threshold, with ", 
        n_samples, 
        " resamples per sample size (sample sizes from ", 
        min(sample_size), 
        " to ", 
        max(sample_size), 
        " were tested). The total amount of resamples/tests is ", 
        nrow(resampled_data), 
        " across all metrics."),
    locations = gt::cells_column_labels(columns = sample_size)
  )
  

```
```
