# Supplementary figures and images for: Power analysis for personal light exposure measurements and interventions
Source: PLoS One. 2024 Dec 11;19(12):e0308768. doi: 10.1371/journal.pone.0308768 (PMC11633969; doi:10.1371/journal.pone.0308768)

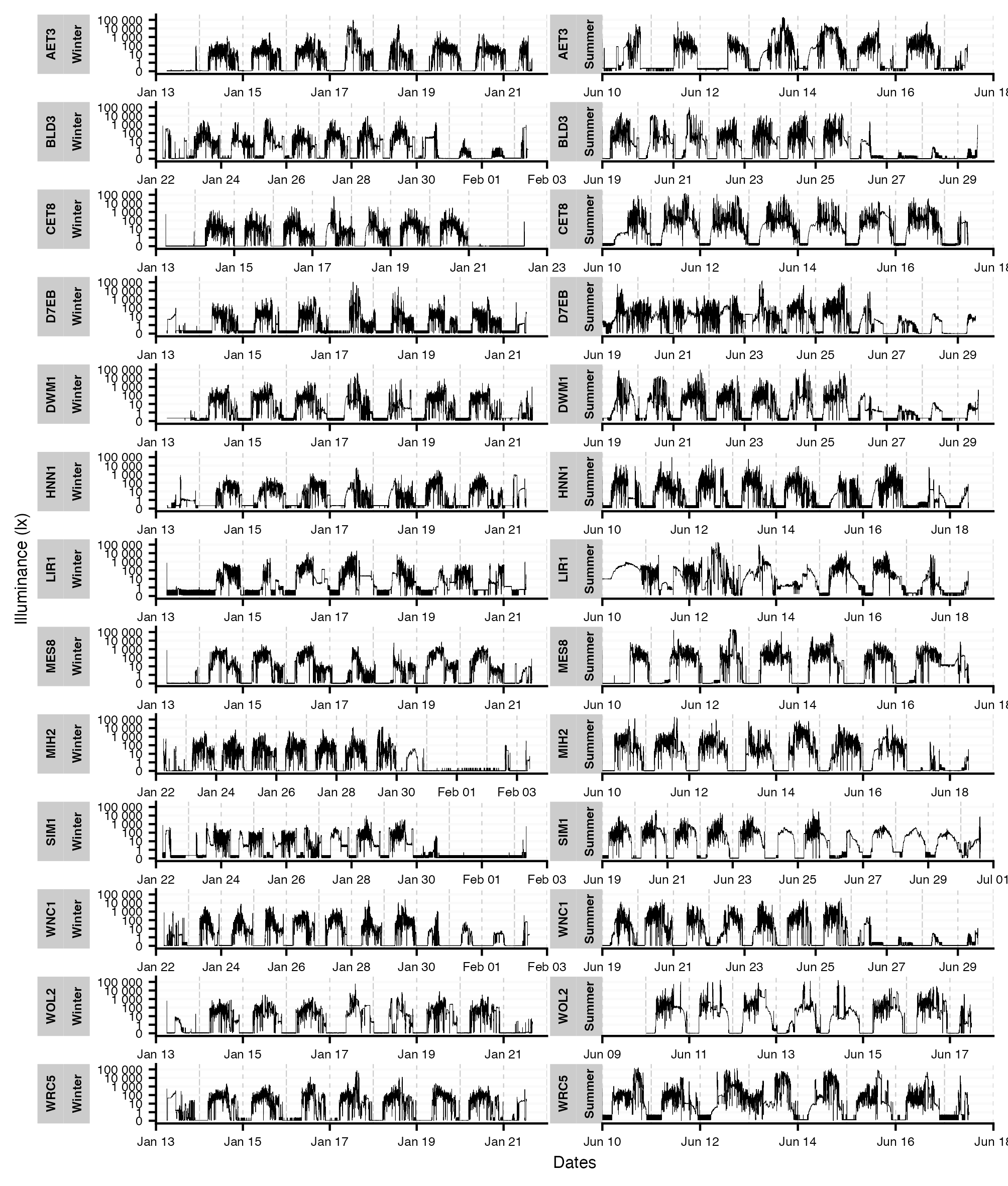

Supplement: S1 Fig — (PNG) [file pone.0308768.s002.png]

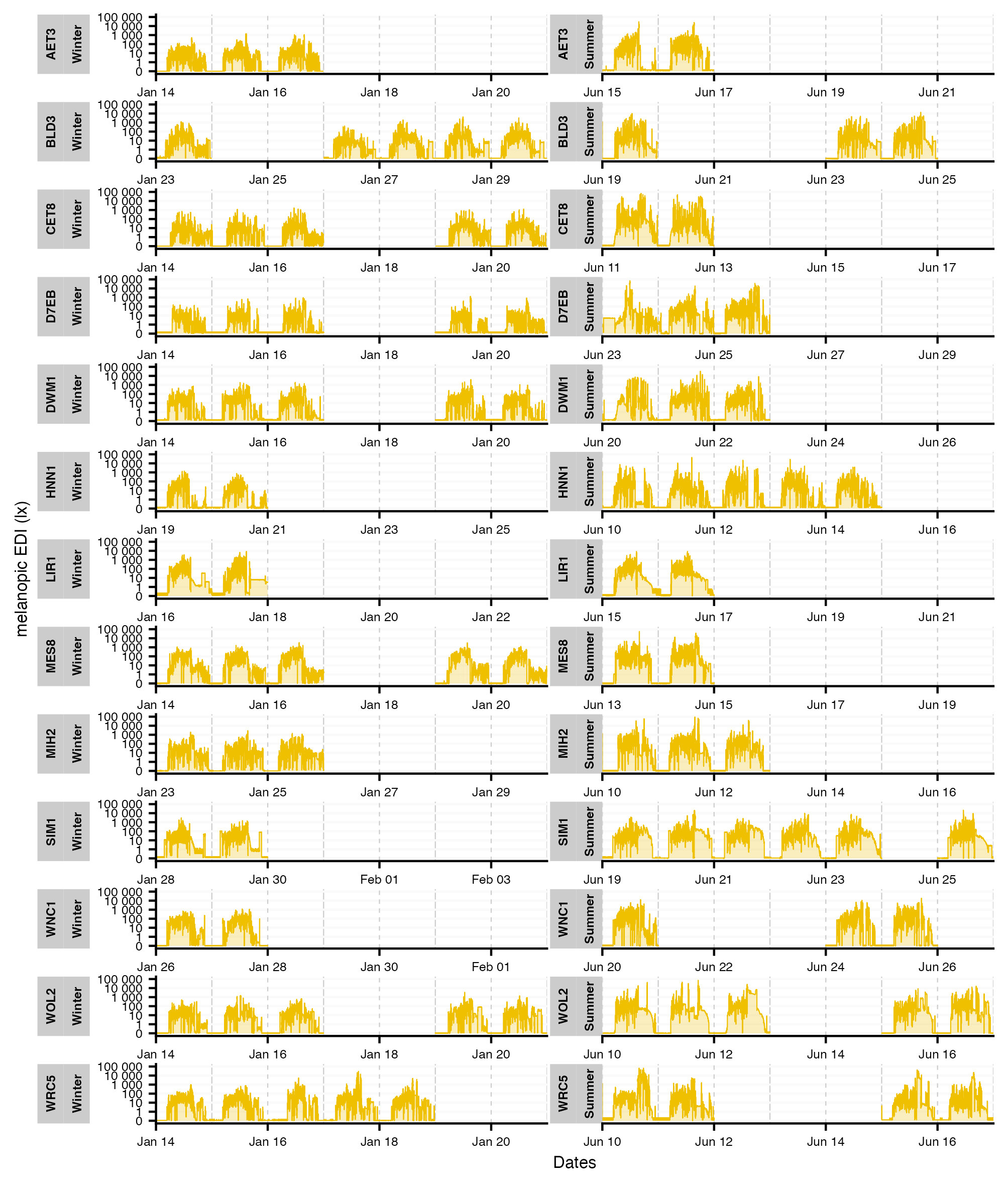

Supplement: S2 Fig — (PNG) [file pone.0308768.s003.png]

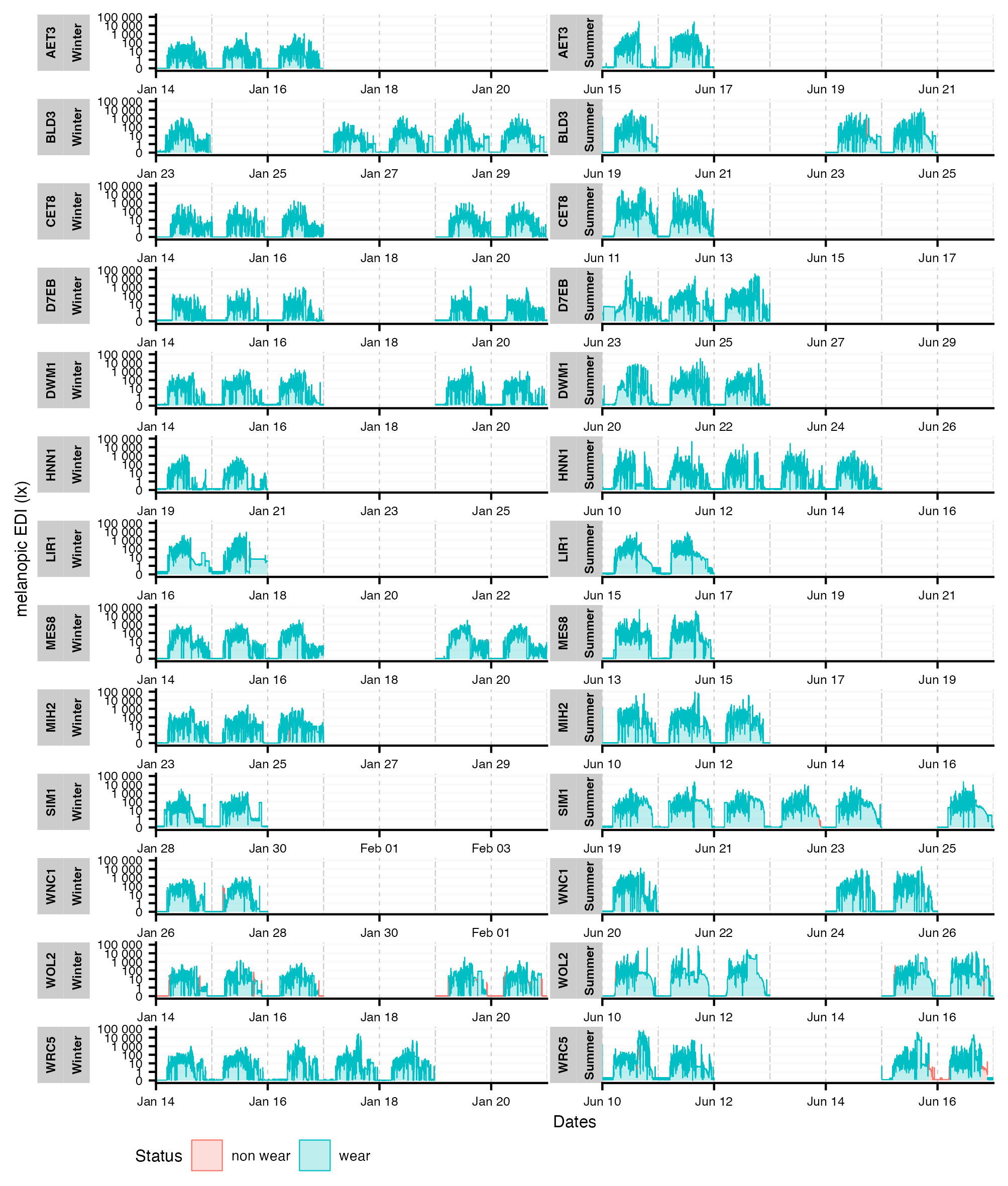

Supplement: S3 Fig — (PNG) [file pone.0308768.s004.png]

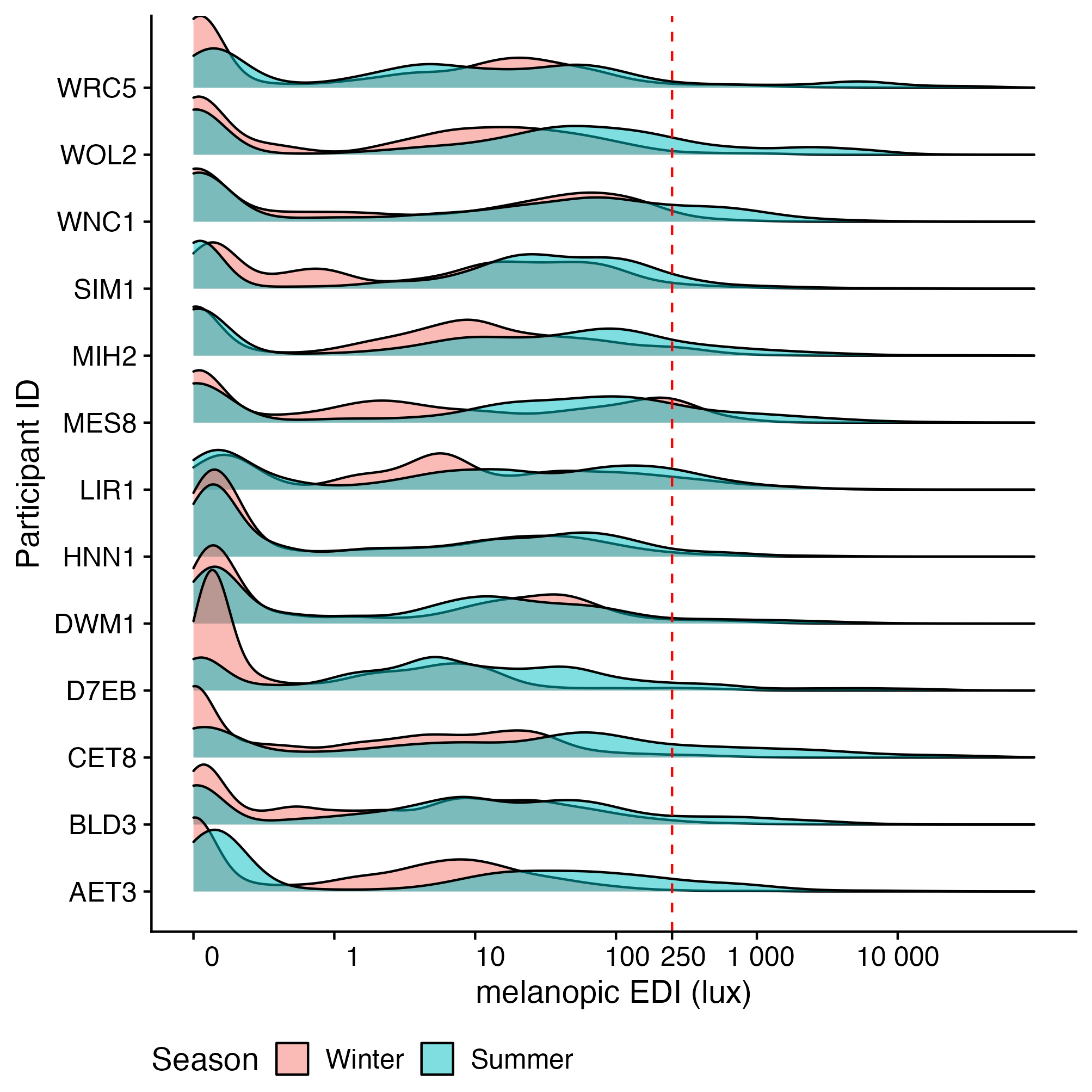

Supplement: S4 Fig — A red dashed line indicates a threshold of 250 lux. (PNG) [file pone.0308768.s005.png]
