# Supplementary material for: Power analysis for personal light exposure measurements and interventions
Source: PLoS One. 2024 Dec 11;19(12):e0308768. doi: 10.1371/journal.pone.0308768 (PMC11633969; doi:10.1371/journal.pone.0308768)
Supplement: Attachment — Submitted filename: Reviewer Report.docx [file pone.0308768.s006.docx]

**Reviewer Report**

**Manuscript Title**:
“Power analysis for personal light exposure measurements and interventions”

**1. Summary of the Manuscript**

The manuscript addresses the challenge of estimating sample size by introducing a hierarchical bootstrapping procedure. This approach effectively handles the complex hierarchical structure of the light exposure data through stepwise resampling and mixed-effects model. The proposed method is applied and validated in twelve summary metrics of light exposure, revealing a significant variability in sample size requirements among different metrics, with some metrics requiring substantially larger sample sizes than others, highlighting the importance of context-specific considerations in experimental design.

2. **Recommendation**

The manuscript presents a valuable and novel method for sample size estimation in light exposure studies. I recommend **major revisions** to improve clarity, provide more detail on the methodology, and expand on the implications of the findings. Providing additional context and examples will enhance the overall clarity and impact of the manuscript.

3. **General Comments**

The manuscript contributes to the field by offering a novel approach for sample size estimation in studies beyond seasonal differences and for different activity patterns. The introduction of hierarchical bootstrapping is a valuable advancement given the complexity of light exposure data. The dataset provided further enhances the manuscript's practical utility. However, additional details and context would strengthen the overall presentation. The manuscript would also benefit from more information on the validation of the method or comparisons with other approaches to fully demonstrate its effectiveness.

4. **Specific Comments**
1) The abstract and introduction set the context well but could more detailed explain why current sample size decisions are inadequate or unrelated to power analysis. More comprehensive comparisons between the proposed method and the existing method would underscore the significance of the new method. For example, the manuscript contrasts the proposed bootstrapping method with the Kumle et al. approach. It would be helpful to provide a quantitive comparison of the strengths and limitations of both methods, particularly in terms of their applicability to different types of datasets or research questions.
2) The authors emphasize using their method on the time series dataset, a clearer explanation of its key advantages over traditional methods on time series data would provide clearer insight into its benefits.
3) The manuscript uses the dataset from early-day shift workers to validate the proposed method, but the limitations of this dataset should be addressed more explicitly.
5. **Minor Issues**
1) The figure captions need to be improved.
2) Consider changing "wearable light loggers" to "wearable light exposure loggers" for clarity.
3) The phrase “a large range of required sample sizes from 3 to >50” could be rephrased to “sample sizes ranging from as few as 3 to more than 50” for better readability.
4) In the power analysis section, the meaning of coefficient beta_i needs to be clarified.
5) The method section needs to be more organized.
